# Recruitment to a Randomized Web-Based Nutritional Intervention Trial: Characteristics of Participants Compared to Non-Participants

**DOI:** 10.2196/jmir.1086

**Published:** 2009-08-26

**Authors:** Melanie A Stopponi, Gwen L Alexander, Jennifer B McClure, Nikki M Carroll, George W Divine, Josephine H Calvi, Sharon J Rolnick, Victor J Strecher, Christine Cole Johnson, Debra P Ritzwoller

**Affiliations:** ^6^Center for Health Communications ResearchUniversity of Michigan School of Public HealthAnn ArborMIUSA; ^5^HealthPartners Research FoundationMinneapolisMinnesotaUSA; ^4^Kaiser Permanente GeorgiaCenter for Health Research SoutheastAtlantaGeorgiaUSA; ^3^Group Health Center for Health StudiesSeattleWashingtonUSA; ^2^Henry Ford HospitalDetroitMichiganUSA; ^1^Institute for Health ResearchKaiser PermanenteDenverColoradoUSA

**Keywords:** Recruitment, Web-based interventions, making effective nutritional choices, Cancer Research Network, CRN, fruits and vegetables, research subject selection, selection, patient, mass screening, Internet, motivation, cultural diversity, health maintenance organizations

## Abstract

**Background:**

Web-based behavioral programs efficiently disseminate health information to a broad population, and online tailoring may increase their effectiveness. While the number of Internet-based behavioral interventions has grown in the last several years, additional information is needed to understand the characteristics of subjects who enroll in these interventions, relative to those subjects who are invited to enroll.

**Objective:**

The aim of the study was to compare the characteristics of participants who enrolled in an online dietary intervention trial (MENU) with those who were invited but chose not to participate, in order to better understand how these groups differ.

**Methods:**

The MENU trial was conducted among five health plans participating in the HMO Cancer Research Network in collaboration with the University of Michigan Center for Health Communication Research. Approximately 6000 health plan members per site, between the ages of 21 and 65, and stratified by gender with oversampling of minority populations, were randomly selected for recruitment and were mailed an invitation letter containing website information and a US$2 bill with the promise of US$20 for completing follow-up surveys. Administrative and area-based data using geocoding along with baseline survey data were used to compare invitees (HMO members sent the introductory letter), responders (those who entered a study ID on the website), and enrollees (those who completed the enrollment process). Generalized estimating equation multivariate and logistic regression models were used to assess predictors of response and enrollment.

**Results:**

Of 28,460 members invited to participate, 4270 (15.0%) accessed the website. Of the eligible responders, 2540 (8.9%) completed the consent form and baseline survey and were enrolled and randomized. The odds of responding were 10% lower for every decade of increased age (*P* < .001), while the likelihood of enrolling was 10% higher for every decade increase in age (*P* < .001). Women were more likely to respond and to enroll (*P* < .001). Those living in a census tract associated with higher education levels were more likely to respond and enroll, as well as those residing in tracts with higher income (*P* < .001). With a 22% (n = 566) enrollment rate for African Americans and 8% (n = 192) for Hispanics, the enrolled sample was more racially and ethnically diverse than the background sampling frame.

**Conclusions:**

Relative to members invited to participate in the Internet-based intervention, those who enrolled were more likely to be older and live in census tracts associated with higher socioeconomic status. While oversampling of minority health plan members generated an enrolled sample that was more racially and ethnically diverse than the overall health plan population, additional research is needed to better understand methods that will expand the penetration of Internet interventions into more socioeconomically diverse populations.

**Trial Registration:**

Clinicaltrials.gov NCT00169312; http://clinicaltrials.gov/ct2/show/NCT00169312 (Archived by WebCite at http://www.webcitation.org/5jB50xSfU)

## Introduction

Web-based behavioral programs can efficiently and effectively disseminate health education to a broad population [1] and allow people to access multimedia information on their own schedule, at their own pace and location, without geographic limitations or the expense of face-to-face interactions [2]. Online tailoring may increase the effectiveness of these programs [3]. Indeed, many health plans and for-profit wellness companies are now offering Web-based health behavior change programs [1], and more attention is now being given to understanding effective recruitment for such Web-based programs [4]. The future effectiveness of Internet-delivered health promotion programs as population-based interventions will depend on their ability to reach and engage a broad variety of users.

While Internet-based programs have the potential to reach millions of people, potential reach is not actual reach [5]. Health plans (health maintenance organizations, HMOs) maintain comprehensive electronic data, making the recruitment process efficient since individuals can be readily identified and targeted by age, gender, diagnoses, and procedures. These databases also allow the research teams to track participation and eventually outcomes related to health and medical care [6,7].

We previously reported on the importance of combining pre- and post-enrollment financial incentives for recruiting and maintaining participation in an online health promotion program [8]. A recent study that compared response rates by gender and racial subgroups to determine incentive combinations that would better recruit a diverse group of healthy adults who were members of a Midwestern HMO to an online health program [8] determined that a combination of a US$2 bill and the promise of US$20 for completing an online follow-up survey was most effective across gender and broad racial/ethnic subgroups. What is not known is the effect of this combination across diverse geographic regions.

Furthermore, to our knowledge, no one has examined who enrolls in online health promotion programs and how the characteristics of these people differ from those who do not participate. This information has important implications for those designing and marketing online health promotion programs; however, data on nonresponders are often unavailable, making such a comparison impossible. Using automated health plan data and geocoding, we were able to examine this issue in the Making Effective Nutritional Choices (MENU) trial, which is an online dietary intervention study funded by the National Cancer Institute and open to members of five US health plans [9].

Based on data from other health promotion programs, both Internet-based and otherwise [10], we hypothesized that women and persons of higher socioeconomic status would be more likely to enroll in MENU [11-13]. This paper reports the results of the MENU recruitment efforts, including a comparison of the demographic characteristics of people who were invited but did not respond (invitees), people who visited the website and entered their log-in ID but did not enroll (responders), and those who were eligible and enrolled (enrollees). We believe our results shed important light on the demographic characteristics of people interested and willing to participate in an online health promotion intervention study and will help inform this burgeoning area of interest.

## Methods

### Setting

We developed an Internet-based program to promote increased intake of fruits and vegetables, “Making Effective Nutritional Choices (MENU),” and tested it among diverse members of five health plans. MENU was a randomized trial conducted in conjunction with the HMO Cancer Research Network (CRN) [9]. The CRN consists of the research programs, enrollee populations, and legacy databases of the participating integrated health care organizations. The goal of the CRN is to conduct research on cancer prevention, early detection, treatment, long-term care, and surveillance [14]. Five of the CRN affiliated health care delivery systems—Group Health in Seattle, Kaiser Permanente Colorado in Denver, HealthPartners in Minneapolis, Henry Ford Health System in Detroit, and Kaiser Permanente Georgia in Atlanta—collaborated with the University of Michigan’s Center for Health Communications Research for the MENU study. The Institutional Review Boards from all participating institutions approved this study prior to data collection.

### Recruitment

For the remainder of the paper, the following terms with associated definitions will apply: Invitees are defined as members of one of the five health plans noted above who were mailed a recruitment letter; responders are defined as any invitee who entered a study ID on the MENU website; enrollees are defined as responders who completed all enrollment steps in the study, which included an online eligibility survey, consent form, and baseline survey.

### Invitees

Using administrative databases, each site identified a random sample of individuals aged 21-65 years at the beginning of the study enrollment period (September 2005) who were current members with at least 1 year enrollment in the respective health plan with no enrollment gaps greater than 90 days at one time. We used diagnostic codes in the health plan databases to exclude from the sample anyone with a medical condition that could be negatively affected by increasing intake of fruits and vegetables. These conditions included current cancer treatment, gastroparesis, neurological conditions, mental health conditions, and use of anticoagulant medications. From potentially eligible members, we drew a random sample of approximately 6000 individuals from each participating health plan, stratified by gender, with a 10% response goal for study enrollment.

At three sites, minority racial/ethnic groups (African American at Sites 2 and 5, and Hispanic at Site 4) were oversampled in order to enhance the enrollment of these populations. The demographic characteristics of the respective health plan memberships are shown in Table 1. At the time this study began, health plans did not prospectively collect data on the race and ethnicity of their members. Race and ethnicity of the respective health plan members were estimated based on various health plan surveys, census data, etc. Approximately 35% of the membership of Site 2 was African American. Only at Site 2 could the race/ethnicity category for members be ascertained from administrative databases; therefore, the sample pool included 60% of the invitees from the African American strata and 40% from “all others”; both categories were stratified by gender. Approximately 32% of the Site 5 enrollment population was African American, and two of its medical clinics had an estimated African American population of 90%. Site 5 oversampled for African Americans by pulling 61% of its sample from these two predominately African American clinics. Part way through recruitment, in response to an overall lower enrollment rate, this site added an additional 887 invitees, using the same oversampling methodology, to the recruitment pool. Approximately 16% of Site 4’s population was Hispanic. This site weighted 50% of its total recruitment by a probable Hispanic indicator. Probable Hispanics were identified with a Latino surname algorithm based on the Passel-Word Spanish surname list utilized by the 1990 Census [15-18] (also Carroll NM et al, unpublished data, 2009).

**Table 1:** Characteristics of the entire membership of participating MENU health plans

	Site 1	Site 2^a^	Site 3	Site 4	Site 5
Total enrollment (× 1000)	480	250	669	373	271
**Age (years), %**					
≤ 24	35	39	37	34	32
25-44	32	35	39	28	33
45-64	22	20	22	26	28
65-74	12	6	1	9	4
≥ 75	11	–	1	6	2
Female, %	53	53	52	51	53
**Race, %**					
White	89	60	85	75	58
African American	4	35	6	6	32
Asian American	5	< 1	5	2	4
Native American	1	< 1	1	1	< 1
Hispanic	1	< 1	3	16	3
Other	0	2	0	1	2
Web access, %	75	73	83	78	79

^a^ Includes only members assigned to staff model medical group (physicians who are employed by the health plan).

**Figure 1:**
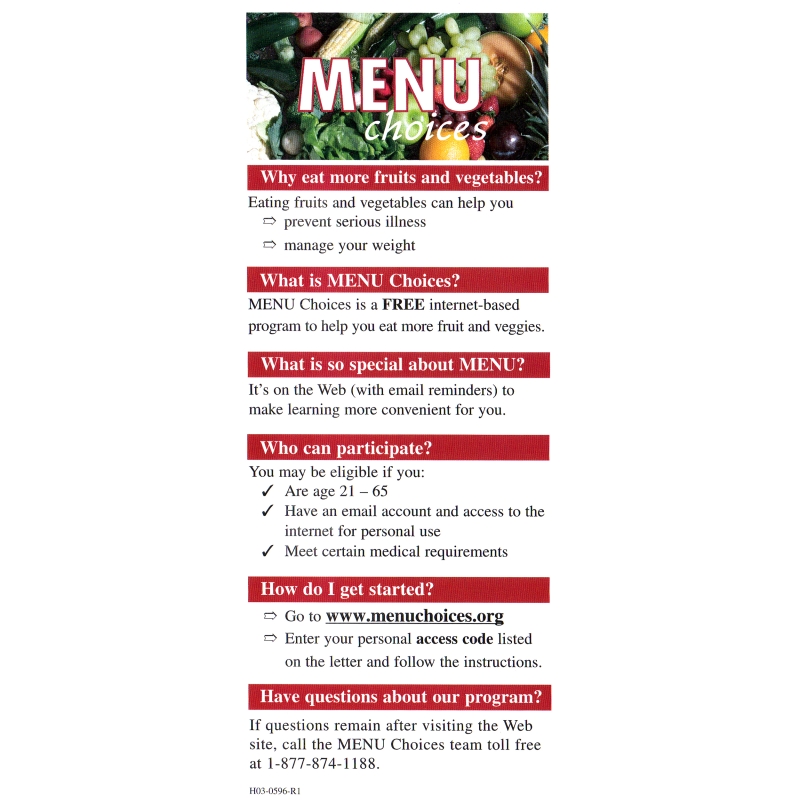
MENU flyer

Invitees were mailed a single introductory letter from each respective site that described the study, was signed by each health plan’s respective investigator, and was postmarked locally using metered postage. This local focus was intended to increase members’ confidence in the study invitation [19,20]. Study-related information, a unique log-in ID, and the name and telephone number of the health plan’s contact person were included in the letter. The letter was accompanied by a colorful 3-inch by 8-inch flyer (Figure 1) inviting recipients to participate in the study and a pre-paid monetary incentive [8]. Both the letter and flyer invited the recipient to “check out” the website and provided the MENU study’s URL and toll-free telephone number. An incentive combination was used based on results from previous work investigating optimal incentives for Web-based health program participation and retention [8]. Each introductory letter contained a US$2 bill as an enrollment incentive and described the US$20 promised incentive that would be sent for completion of each of the three online follow-up surveys, over the course of 1 year. All study materials were provided in English only. Since the population at Site 4 may have included a large number of non-English-proficient invitees, the introductory letter from this site included Spanish text explaining that the MENU program was being offered in English only.

### Responders

Those interested in participating logged on to the study website and were asked to answer questions (pre-consent) and then to complete a short survey that confirmed eligibility status. The eligibility survey included 9 to 12 questions, depending on personal tailoring, and included questions to confirm health plan membership status, age, accessibility to the Internet for personal use, frequency of use of a personal email address, and history and treatment of certain health conditions. Eligibility was restricted to those who reported having access to the Internet for personal use, who had a working email account that they used at least once a week, and who did not have a health condition that conflicted with eating fruits and vegetables.

Responders found to be eligible were presented with an online informed consent form. The process asked members to read the consent information and click “I agree” after each page before being able to move to the next page of the consent form. The toll-free telephone help number and a “help” link appeared at the bottom of each page. The final screen provided the option to print a paper copy of the consent form.

### Enrollees

Following completion of the consent process, the website prompted the responders to provide email and postal contact information so that incentives and email reminders could be sent as part of the intervention protocol. The email address was verified through a reply email sent automatically from the study website server. Once the email address was verified, the online baseline survey was available for completion. It was comprised of approximately 70 questions and took approximately 25 minutes to complete. Participants were considered “enrolled” after they completed the survey. The Web programming allowed participants to complete the survey over several sessions, if interrupted. Responders were given 28 days to begin the baseline survey and 28 days to complete the survey once started. During this time, up to four automated email reminders were sent, one every three or four days, to persons with incomplete surveys. After the final 28 days expired, the survey was closed to those not completing the enrollment process. Enrollees were those participants completing all steps of enrollment.

### Measures

Age, gender, and residential address were obtained from administrative databases for each invitee. We employed geocoding techniques to the residential address to create area-based proxies for income and education as socioeconomic variables for each invitee. Geocoding was performed using MapMarker Plus and 2000 Census data. Each participant’s address was mapped to a census tract and the corresponding median household income and proportion of the census tract attaining various educational levels. Indicator variables were created for each invitee, with cut points at median household income and post–high school versus less educational levels. Race, ethnicity, and other demographic and socioeconomic variables were obtained by self-report from enrollees in the baseline survey.

### Statistical Analysis

The numbers of invitees, responders, and enrollees were tabulated. Descriptive statistics were computed to characterize demographics and geocoded information for each group. Statistical significance of differences was tested using the Wilcoxon rank sum or the chi-square test. The protected least significant difference approach to multiple comparisons was used to compare enrollment rates among the sites.

The associations between age, gender, and census-derived household income and education indicators, and between response and enrollment, were assessed using generalized estimating equation (GEE) multivariable logistic regression. GEE was used to take into account clusters defined by site. Customary residual and influential statistics were examined to assess model fit and evaluate outliers. Analyses were conducted using SAS 9.1 [21].

## Results

Characteristics are presented for each participating health plan population (Table 1). Across the five study sites, the estimated proportion of African Americans ranged from 4% to 35%, with an overall average of 12.5%. The proportion of Hispanic members ranged from < 1% to 16%, with an overall average of 4.5%.

**Figure 2:**
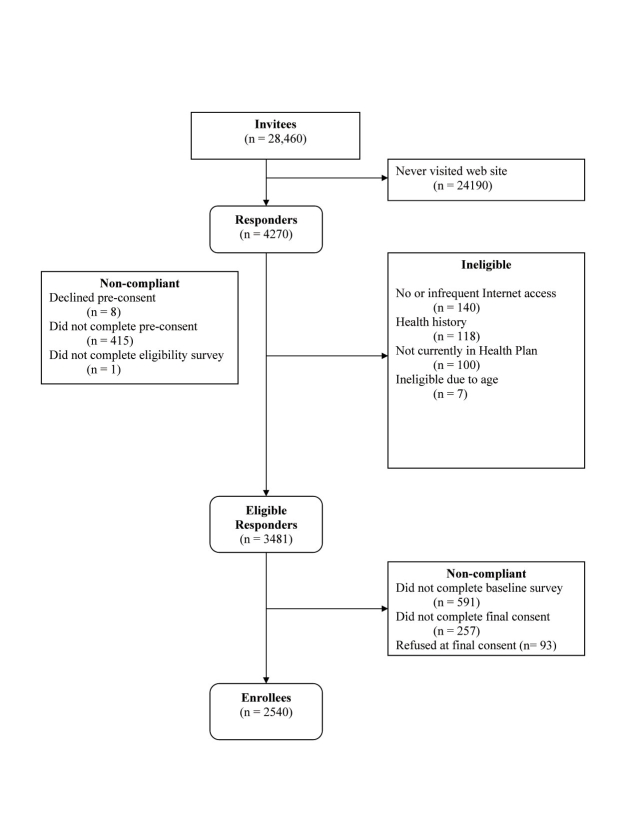
Recruitment flowchart for MENU

Figure 2 indicates the flow of participants from invitation to enrollment. A total of 28,460 adults from each of the five health plans were invited to participate; 15% (n = 4270) responded to the introductory letter, visited the website, and entered their unique log-in ID (responders). Of these, 18.5% (n = 789) failed to complete the enrollment process. Reasons for dropoff included not completing the pre-consent or eligibility questions (n = 424, 54%), ineligibility due to no or infrequent Internet access (n = 140, 17.7%), ineligibility due to medical history (n = 118, 15.0%), or ineligibility due to not being currently enrolled in the health plan (n = 100, 12.7%). Out of the eligible responders (n = 3481), a total of 941 (27.0%) did not enroll. Additional details on the reasons for dropout are provided in Figure 2. In total, 2540 (8.9% of invitees) enrolled.

Characteristics of the invitees, responders, and enrollees derived from geocoding, administrative data, or self-report are summarized in Table 2. While 47.8% of the invitees lived in a census area defined as having greater than 63% completing high school, 56.3% of the responders and 58.7% of the enrollees lived in such areas. Similarly, 40.1% of invitees mapped to a census area median household income of at least US$52,250, and half of the responders and enrollees mapped to this higher income level. Comparing those enrolled to those invited, enrollees were more likely to be from households with higher education (10.9% vs 7%, *P* < .001) and income (11.1% vs 7.4%, *P* < .001). Women represented 50.2% of invitees and made up 61.4% of responders and 64.8% of enrollees. On average, responders were slightly older than invitees and enrollees (46.0 years vs 44.3 years and 45.5 years, respectively.) The percentage of enrollees compared to invitees varied by site, with the highest enrollment seen in Site 1 (13.1%, *P* < .001). Finally, the enrollees were comprised of 22.3% (n = 566) African Americans and 7.6% (n = 192) Hispanics.

**Table 2:** Descriptive characteristics of invitees, responders, and enrollees in MENU^a^

Characteristic	Invitees (n = 28,460)	Responders (n = 4270)	Enrollees (n = 2540)	Enrolled/ Invited^c^
	No. (%)	No. (%)	No. (%)	%
**Census-Derived Data**^**b**^				
** Education**				
< 63% high school or greater	13,622 (52.2)	1705 (43.7)	957 (41.3)	7
≥ 63% high school or greater	12,461 (47.8)	2195 (56.3)	1360 (58.7)	10.9
** Median household income**				
<US$52,250	15,621 (59.9)	1994 (51.1)	1154 (49.8)	7.4
≥ US$52,250	10,462 (40.1)	1906 (48.9)	1163 (50.2)	11.1
				
**Administrative Data**				
** Gender**				
Female	14,298 (50.2)	2621 (61.4)	1645 (64.8)	11.5
Male	14,162 (49.7)	1649 (38.6)	895 (35.2)	6.3
** Health plan site**				
Site 1	4053 (14.2)	845 (19.8)	532 (20.9)	13.1
Site 2	6372 (22.4)	930 (21.8)	520 (20.5)	8.2
Site 3	4332 (15.2)	712 (16.7)	456 (18.0)	10.5
Site 4	5751 (20.2)	805 (18.9)	514 (20.2)	8.9
Site 5	7952 (27.9)	978 (22.9)	518 (20.4)	6.5
				
**Survey Data**				
** Race**				
White/other	--	--	1935 (77.4)	--
African American	--	--	566 (22.6)	--
** Hispanic ethnicity**				
Yes	--	--	192 (7.6)	--
No	--	--	2325 (92.4)	--
** Highest level of education**				
≤ High school or vocational tech	--	--	397 (15.7)	--
Some college	--	--	855 (33.8)	--
College degree	--	--	663 (26.2)	--
Post-grad	--	--	617 (24.4)	--

^a^ Some variables had missing data; thus, numbers may not equal total.

^b^ A total of 4274 (15.0%) invitees, 564 (13.2%) responders, and 333 (13.1%) enrollees had addresses that could not be mapped to the census data.

^c^ Enrollee versus invitee associations were all statistically significant (*P* < .001).

Table 3 presents results from a GEE logistic regression model that was used to evaluate the association between invitee age, gender, and indicator variables for census area median household income and education and visiting the MENU website and entering the study ID. Age, gender, and census area household income and education were all statistically significantly associated with being a responder. For every decade increase in age, a 0.89 decrease in odds of responding was observed (95% CI 0.82-0.96, *P* = .002).

**Table 3:** Adjusted odds ratios for predicting a response to the MENU letter among invitees by age, gender, and census area income and education

Variable	Odds Ratio^a^	95% CI	*P*
Age (decade)	.89	.82, .96	.002
Female	1.40	1.19, 1.63	< .001
Higher census area income	1.11	1.01, 1.23	.04
Higher census area education	1.17	1.05, 1.29	.004

^a^ All odds ratios adjusted for other variables in table.

Table 4 describes the results from a GEE logistic regression model predicting enrollment. All characteristics included in the model were statistically significantly associated with enrollment status, with female gender having the strongest association (95% CI 1.63-2.16, *P* < .001).

**Table 4:** Adjusted odds ratios for predicting enrollment of invitees by age, gender, and census area income and education

Variable	Odds Ratio^a^	95% CI	*P*
Age (decades)	1.10	1.06, 1.13	< .001
Female	1.88	1.63, 2.16	< .001
Higher census area income	1.32	1.19, 1.46	< .001
Higher census area education	1.36	1.10, 1.68	.004

^a^ All odds ratios adjusted for other variables in table.

We did not have race/ethnicity invitee data from most of the participating health plans, and therefore could not use them as a predictor of response or enrollment. Our attempts to oversample minority populations are shown in the enrollment in Table 5, reflecting the underlying health plan minority distribution. While the estimated proportion of African Americans varied across all five CRN health plans (see Table 1), oversampling resulted in the MENU study enrollees being comprised of 22.3% (n = 566) self-identified African Americans. Across all five sites, the proportion of Hispanics who enrolled was 7.6% (n = 192).

**Table 5:** Race and ethnicity of MENU enrollees

	Site 1 No. (%)	Site 2^a^ No. (%)	Site 3 No. (%)	Site 4^a^ No. (%)	Site 5^a^ No. (%)	Total No. (%)
African American	8 (1.5)	230 (44.2)	13 (2.9)	14 (2.7)	301 (58.1)	566 (22.3)
White	469 (88.2)	248 (47.7)	413 (90.6)	369 (71.8)	169 (32.6)	1668 (65.7)
Other	47 (8.8)	41 (7.9)	29 (6.4)	110 (21.4)	40 (7.7)	267 (10.5)
Unknown	8 (1.5)	1 (0.2)	1 (0.2)	21 (4.1)	8 (1.5)	39 (1.5)
						
Hispanic	21 (4.0)	7 (1.4)	5 (1.1)	146 (28.4)	13 (2.5)	192 (7.6)
Non-Hispanic	506 (95.1)	508 (97.7)	447 (98.0)	367 (71.4)	497 (96.0)	2325 (91.5)
Unknown	5 (0.9)	5 (1.0)	4 (0.9)	1 (0.2)	8 (1.5)	23 (0.9)

^a^ Sites oversampling for diverse populations: Site 2 and Site 5 oversampled for African Americans, and Site 4, for Hispanics.

## Discussion

This study describes the results of a recruitment effort to enroll members of five health plans across the United States into a Web-based behavioral intervention trial. A significant mailing volume was needed to achieve our target enrollment, with a final enrollment rate of nearly 9% of invitees. This enrollment rate is consistent with other Web-based enrollment efforts [10]. Site enrollment rates varied, with overall sampling yielding a diverse group of men and women and minorities. Similar to findings of Oenema et al [22], women made up two-thirds of the enrollees. In contrast to other findings, this health promotion program appealed to people equally across the education spectrum. Enrollees were equally divided between college completers, graduate school completers, and those with less than college graduate education. Older invitees were less likely to respond to the invitation to visit the website, but were more likely to enroll in the study. This suggests that younger invitees were open to investigating a Web-based study, but consistent with findings by Verheijden and colleagues [23], the older responders actually took the step and enrolled into the Web-based behavior change program.

Of those who enrolled, there were significantly more women; enrollees were generally older and non-Hispanic white and resided in census areas of higher educational and income levels. The enrollment of more women than men was consistent with higher female enrollment in other dietary intervention programs. Women tend to be the shoppers and food preparers of families [11-13,24].

By oversampling minority health plan members at three of the five sites, we were able to enroll a diverse cohort that was over 22% African American and almost 8% Hispanic. While the proportion of overall minority enrollees varied by site, oversampling doubled the proportion of participating African American and Hispanic members relative to their underlying populations, as noted in Table 1, improving the generalizability of the final study outcomes.

Almost 85% of invitees never investigated the website in response to the invitation letter. Efforts were made to encourage letter opening and reduce the appearance of “junk mail,” including using a more business-style envelope with metered postage and a recognizable affiliation (HMO) in the return address [25]. These results may partially reflect self-selection since eligibility requirements were included in the invitation. These low recruitment numbers may reflect a lack of awareness of inadequate dietary patterns [22] and perceived difficulty of or disinterest in increasing fruit and vegetable consumption [26].

Given the large number of invitees who did not even view the website, we assume that there were significant barriers to study enrollment. The 12-month, longitudinal MENU study might have imposed too much time burden on invitees. Additionally, a proportion of invitees probably did not have convenient access to both the Internet and an email account on a weekly basis, even though the number of Americans using the Internet has grown to 79% [27]. Invitees were initially given a US$2 bill in the introduction letter and told that they would receive a higher dollar amount after completion of each of the 3-, 6-, and 12-month surveys [8]. It is possible that these invitees needed some further incentive to continue on to enrollment, including availability of a brief portion of the website program or limited access to it.

Nearly one-third of those presumably eligible who visited the website to read about the study decided not to pursue participation. A respondent viewing the website was met with requests to complete an eligibility survey, give consent, set up a contact account (which included providing home and email addresses and telephone number), and respond to emailed requests to complete a lengthy baseline survey. Completing the lengthy consent and the baseline survey was required for enrollment and access to the study’s intervention website. The consent process required by the Research Ethics Board presented full details on expectations and time frames for participation in this 12-month study. The consent form covered several pages, and skipping pages to the end was not possible. While we simplified the consent form to meet the 7th grade reading level recommendation and used bullets and numbering to aid reading, this Web-based consent “contract” might have proved daunting, particularly to those who had been merely curious about the program requirements and interested in the offered incentives. Future studies need to account for this barrier and consider the enrollment completion rate in determining invitee sample size. In addition, the baseline survey that was also required for enrollment was quite lengthy and could have been a barrier to completing enrollment.

### Limitations and Strengths of the Study

Limitations of this study include relatively limited knowledge of the individual characteristics of the target population. Further, the geocoding yielded low resolution of income and education. There are other variables associated with likelihood of enrolling, for example race and ethnicity, which we could not or did not assess. Practical human subject limitations precluded contacting nonrespondents to elucidate further reasons for not enrolling.

Strengths of this study include a large and diverse target population representing five geographic regions and oversampling of minority members. We recruited from a known sample of potential participants, used health system administrative data to identify age and gender of invitees, and used personal ID access codes to track the Web sign-on information for respondents. Another strength includes our ability to measure response to an online health promotion intervention program, acknowledging the relatively low numbers of those programs currently available [28]. Also, by stratifying invitees by gender, we were able to measure the response rate of men and women.

### Conclusions

Web-based interventions have vast potential to reach virtually anyone with Internet access [29], either through work or personal computers. While only 9% of those invited went on to enroll in MENU, we demonstrated that a mailed invitation letter and online enrollment provided a efficient and relativity successful mode to recruit diverse participants to a Web-based health promotion study. The majority of our enrollees were women, with proxy variables demonstrating relatively high household socioeconomic status. However, through oversampling, minority members enrolled at a higher rate than the membership percentage across the combined health plans. More research and reporting of response and enrollment rates in Web-based research, defining various age, gender, racial and ethnic groups, is needed to enhance the recruitment to eHealth interventions in order to expand participation across diverse populations. Such reporting for Web-based programs designed for other lifestyle interventions to prevent and treat cancer and chronic diseases would greatly enhance future utilization of this medium by all racial and ethnic populations.

## Abbreviations

CRN: Cancer Research Network
HMO: health maintenance organization
MENU: Making Effective Nutritional Choices
